# The Tr. Muriate Ferri in Nasal Polypus

**Published:** 1859-09

**Authors:** J. H. Reeder

**Affiliations:** Lacon, Ill.


ARTICLE IV.

### THE TR. MURIATE FERRI IN NASAL POLYPUS.

BY J. H. REEDER, M.D., OF LACON, ILL.

I do not remember to have seen the above article recommended for the destruction of nasal polypus: if it has been used by any one, an account of it has escaped my notice.

In November last, a little girl, aged about ten years, presented herself at my office, with a polypus of large size, of the gelatinous variety, occupying the left nasal cavity, completely obstructing the cavity, producing considerable deformity by enlargement of that side of the nose.

I attempted its removal by the use of the ordinary polypus forceps, but owing to the smallness of the external nasal opening, they could not be introduced sufficiently high to grasp the root of the tumor. Pretty free hemorrhage and a good deal of pain to the patient was the only result of my endeavors to remove it. To suppress the former, I injected the nostril with tr. mur. ferri, diluted with water, and then forced a piece of moistened sponge into the nostril with the view of dilating it, and told my patient to return in a couple of days, when I hoped to be able to remove the polypus without much difficulty.

The next morning she called, and, much to my surprise, informed me that soon after she arose from her bed the whole mass had escaped from the nostril posteriorly, and had been thrown out on the floor, a semi-fluid opaque mass. On removing the sponge, I found the cavity perfectly free, not a vestige of the polypus remaining. I directed her to throw up the cavity a preparation of the same, much diluted, for a week or two, every alternate day, which she did. No symptom of return has been noticed up to this time.

The second and last case that I have had the opportunity of treating was Mr. Fairbanks, of this city, aged between fifty and sixty years, who has had polypus in both nostrils for more than ten years, and for that length of time has not been able to force a particle of air through the passages, and consequently has been deprived of the senses of taste and smell. His sight was also seriously affected, being, at the time of his coming to me, nearly blind.

I applied the tr. mur. ferri, very nearly full strength, by saturating the sponge of a small probang, and forcing it as far up the nostril as was practicable, repeating the operation every alternate day. In about a week, both nasal cavities were perfectly free from all obstructions. There has been no return of the polypus to my knowledge. He has recovered the power of tasting and smelling; his eyes have also materially improved.

